# Differences in hydrolytic enzyme activity accompany natural variation in mature aleurone morphology in barley (*Hordeum vulgare* L.)

**DOI:** 10.1038/s41598-018-29068-4

**Published:** 2018-07-23

**Authors:** Matthew K. Aubert, Stewart Coventry, Neil J. Shirley, Natalie S. Betts, Tobias Würschum, Rachel A. Burton, Matthew R. Tucker

**Affiliations:** 1 School of Agriculture, Food and Wine, Waite Research Institute, The University of Adelaide, Glen Osmond, SA Australia; 2 Australian Research Council Centre of Excellence in Plant Cell Walls, the University of Adelaide, Adelaide, Australia; 3 State Plant Breeding Institute, University of Hohenheim, Stuttgart, Germany

## Abstract

The aleurone is a critical component of the cereal seed and is located at the periphery of the starchy endosperm. During germination, the aleurone is responsible for releasing hydrolytic enzymes that degrade cell wall polysaccharides and starch granules, which is a key requirement for barley malt production. Inter- and intra-species differences in aleurone layer number have been identified in the cereals but the significance of this variation during seed development and germination remains unclear. In this study, natural variation in mature aleurone features was examined in a panel of 33 *Hordeum vulgare* (barley) genotypes. Differences were identified in the number of aleurone cell layers, the transverse thickness of the aleurone and the proportion of aleurone relative to starchy endosperm. In addition, variation was identified in the activity of hydrolytic enzymes that are associated with germination. Notably, activity of the free fraction of β-amylase (BMY), but not the bound fraction, was increased at grain maturity in barley varieties possessing more aleurone. Laser capture microdissection (LCM) and transcriptional profiling confirmed that *HvBMY1* is the most abundant *BMY* gene in developing grain and accumulates in the aleurone during early stages of grain fill. The results reveal a link between molecular pathways influencing early aleurone development and increased levels of free β-amylase enzyme, potentially highlighting the aleurone as a repository of free β-amylase at grain maturity.

## Introduction

Barley (*Hordeum vulgare* L.) is recorded as one of the first agricultural crops to be domesticated^1^ and is a major food source in both Asia and northern Africa. The highest economic value for the crop is in its use as a malting grain for whisky and beer production^2^. Extensive worldwide cultivation has led to the development and identification of over 460,000 barley accessions, including cultivars, landraces, breeding lines and wild *Hordeum* relatives^3^. Coupled with a diploid sequenced genome^4,5^, these genetic resources provide excellent opportunities to study the fundamental details of barley growth and development, with potential to tailor barley varieties for specific end uses.

Barley grain contains many key nutrients, antioxidants and dietary fibres that benefit the human diet^6–8^. Most of these nutrients accumulate in the endosperm, a filial tissue that supports embryonic growth in addition to providing physical protection during seed development^9^. The endosperm consists of three main cell types - the endosperm transfer cells, starchy endosperm and aleurone layer - each of which confer different biological functions during grain maturation and seed germination. Endosperm development begins after fertilisation of the central cell within the embryo sac^10^, when successive nuclear divisions without cytokinesis lead to the formation of a nuclear syncytium. This mass of nuclei begins to cellularise at the embryo sac periphery at approximately 5 days post anthesis (DPA). The aleurone first appears as a single layer at approximately 6–9 DPA and divides to form multiple layers by around 12–15 DPA. At maturity, the aleurone layers separate the mass of inner starchy endosperm from outer maternal layers, which include the nucellar epidermis, integuments and pericarp. The aleurone cells display a cuboid shape with reinforced cell walls^11^ that are enriched in phenolic acids and polysaccharides such as arabinoxylan^12–14^. During germination, the embryo releases gibberellic acid (GA), which translocates to the aleurone where it induces the transcription of genes encoding hydrolytic enzymes^15^. Enzymes, such as 1,3;1,4-β-glucanase (β-glucanase), α-amylase and β-amylase, are released to catalyse the breakdown of cell wall polysaccharides and starchy energy reserves that are essential for germination and the production of malt for brewing^16^. β-glucanase hydrolyses 1,3;1,4-β-glucan, which is the predominant cell wall polysaccharide present in barley endosperm, α-amylase cleaves internal amylose and amylopectin residues, and the β-amylase exo-hydrolase liberates maltose from the non-reducing end of starch molecules^16^. While α-amylase appears to be transcribed and translated *de novo* during germination, β-amylase is transcribed and translated during grain development^17^. Some of the β-amylase enzyme is present in a free form, while most is present in an inactive bound form, purportedly linked through protein bridges to starch molecules^18–20^.

Seeds from mutants showing defects in aleurone development are often shrunken or misshapen^21^. However, natural differences in aleurone layer number and structure have been observed between cereal species. Cereal grains from species such as maize (*Zea mays* L.) and wheat (*Triticum aestivum* L.) have a single layer of aleurone cells while the barley aleurone is multilayered^22^. Intra-species variation has been found between barley cultivars, and several QTL were identified in an Erhard Frederichen × Criolla Negra population that influence the number of aleurone layers^23^. Genes such as *naked endosperm1*, *supernumerary aleurone layer1*, *defective kernel1* and *crinkly4* influence aleurone development in maize^24–27^, but whether similar genes influence variation in barley aleurone development has yet to be reported. Moreover, the significance of having more or fewer aleurone layers on seed development or germination, particularly in the context of barley, remains unclear.

In this study, 33 barley genotypes were surveyed to identify natural variation in aleurone phenotypes. A method was developed to measure features of the aleurone in mature grain based on UV-autofluoresence of the thick aleurone walls, and to assess correlations with wholegrain traits. Selected genotypes were examined in greater detail to assess the relationship between the aleurone and hydrolytic enzyme activities. Finally, transcriptional profiling of fresh and laser micro-dissected grain tissues was used to ascertain when and where key germination-related genes are transcribed during grain development.

## Materials and Methods

### Plant material

A University of Adelaide (UA) barley diversity panel of 33 genotypes was grown in the field at Charlick, SA, in 2013. A partially overlapping set of genotypes was grown at Gooloogong, NSW, in 2015 and grain was obtained from the National Variety Trials (NVT; www.nvtonline.com.au). The UA panel was chosen to reflect a diverse array of genetic stocks and row-types (Table S1), and consists of both 2-row (n = 30) and 6-row (n = 3) spring genotypes and breeding lines. Grain samples were sieved using a 2.5 mm screen to remove broken grain, long awns and foreign material prior to analysis. The majority of intact grain are retained using this method, allowing analyses to be performed on grains of varying sizes and shapes.

### Grain sectioning and imaging

Mature grain were cut into quarters and fixed overnight in TEM fix (0.25% glutaraldehyde, 4% paraformaldehyde, 4% sucrose in phosphate buffered saline). Samples were rinsed with phosphate buffered saline (3 × 4 hour washes) and then dehydrated in an ethanol series (3 × 8 hours in 70%, 80%, 90%, 95% and 100%). This was followed by an overnight infiltration in a 50:50 mix of 100% ethanol/LR White resin, and 3 changes of pure LR White resin for 8 hours each. Infiltrated specimens were transferred to gelatin capsules in fresh LR White resin, covered with lids and polymerized in a 60 °C oven for at least 48 hours. Sections were prepared at a thickness of 1 μm using a Reichert Ultracut ultramicrotome (Leica, Wetzlar, Germany). For the anatomical study of aleurone cells, sections were stained with 0.01% (w/v) Toluidine Blue and viewed using brightfield microscopy, or 0.001% (w/v) Calcofluor White and viewed using Zeiss Filter set 47 (BP 436/20, FT 455, BP 480/40; blue staining in Fig. S3) and Filter set 46 (BP 500/20, FT 515, BP 535/30; false coloured red in Fig. S3) on a Zeiss M2 AxioImager equipped with DIC optics and an Apotome.2 (Zeiss, Germany).

**Figure 1:**
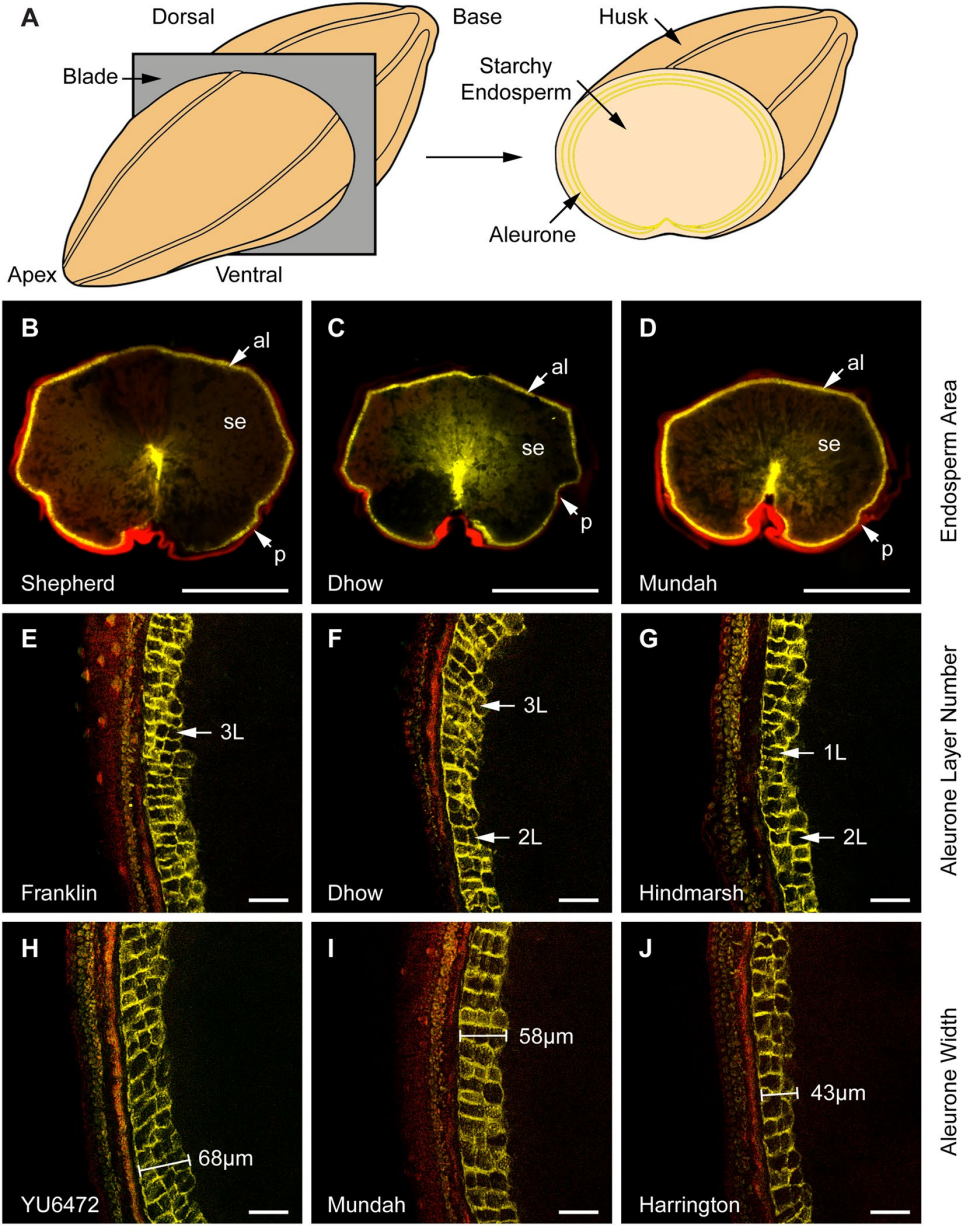
Representation of the transverse sectioning process used to image barley aleurone tissue by fluorescent microscopy. (**A**) Schematic representation of barley grain sectioning prior to microscopy. The different tissue layers are indicated. (**B**–**D**) Wholegrain transverse sections viewed at 1× magnification using Zeiss Filter sets 46 (false-coloured red) and 49 (DAPI; false-coloured yellow). The panels show grain exhibiting differences in transverse starchy endosperm area in decreasing order. The pericarp/husk (p), starchy endosperm (se) and aleurone (al) tissues are indicated. Scale bar = 1 mm. (**E**–**G**) Magnified views of the aleurone layers at 20× magnification using a Zeiss Apotome.2. Panels are arranged in decreasing order based on the average number of aleurone layers. Stacks of 3, 2 and 1 aleurone cell layers (L) are indicated. Scale bar = 50 µm. (**H**–**J**) Examples of grain showing differences in aleurone width at 20× magnification, arranged in decreasing order. Scale bar = 50 µm. Genotype names are indicated in each panel.

To observe the aleurone in fresh samples, mature grain were bisected by hand (transversely) using a reinforced single-edge razor blade (ProSciTech, Australia) and adhered to a microscopy slide using Blu-Tack® (Bostick, Australia) with the flat midpoint of the grain facing upwards. Between 3 and 10 grain from each cultivar were imaged using a Zeiss M2 AxioImager with an attached AxioCam MrM camera (Zeiss, Germany). Zeiss Filter set 46 (BP 500/20, FT 515, BP 535/30) was used to view pericarp and husk autofluorescence (false coloured red in Fig. 1) and Filter set 49 (G365, FT395, BP445/50) was used to view aleurone wall autofluoresence (false coloured yellow in Fig. 1). Images were processed using ZEN 2012 software (Zeiss, Germany).

Grain measurements were also recorded using ZEN 2012 software (Zeiss, Germany; Fig. S1). Transverse endosperm area was measured by tracing the outline of whole endosperm, while aleurone area was calculated by subtracting the starchy endosperm area from the total endosperm area. Aleurone proportion was measured by calculating the aleurone area as a percentage of the total transverse endosperm area. Aleurone layer number was recorded as an average where, in each section of barley grain, a maximum and minimum layer number was recorded at dorsal, left and right positions. Similarly, aleurone width was measured as the distance from the edge of the endosperm to the innermost autofluorescent aleurone cell wall.

### Wholegrain phenotypic analysis

Barley grain weight and dimensions were determined using a SeedCount^TM^ SC4 (Seed Count Australasia, Condell Park, Australia), following manufacturer’s instructions. Single grain hardness, moisture content and diameter of 300 grain were analysed using a Single Kernel Characterisation System, SKCS 4100 (SKCS; Perten Instruments, Springfield, IL), following the manufacturer’s instructions.

### Grain germination and sample preparation for enzyme assays

Grain from selected barley genotypes was placed at 37 °C for two days to remove residual moisture. Dry grains were subsequently sprinkled onto 10 cm diameter No 1. Whatman® filter paper disks (×2), placed in a 10 cm diameter Petri dish and soaked with 4 mL sterilised water. For the germination assay, 70 sample grains were plated alongside 30 control Navigator grain. For the enzyme assays, 30 sample grains were used alongside 70 sacrificial Sloop and Navigator grains for standards and water saturation balances. The Petri dishes were sealed with Parafilm® (Bemis, USA) then placed in an incubator at 20 °C in the dark for 6, 12, 24, 48 or 96 hours. For the germination assay, plates were removed and scored visually to determine the frequency of grain germination at each time point. For the enzyme assays, grain were removed from the incubator and all 30 germinating grains placed into 10 mL tubes (for each variety) and freeze dried for 96 hours to remove residual moisture. Mature grain and dried germinated grains were ground to flour with a Retsch MM400 Mixer Mill (Retsch GmbH, Haan, Germany).

### Hydrolytic enzyme assays

Enzyme assays were performed on both mature grain and dried germinated grain flour, respectively, using downscaled methods (approximately four-fold) from Megazyme (Ireland). The β-Glucanase Assay Kit (K-MBGL)^28^, the α-Amylase Assay Kit (K-CERA)^29^ and the Betamyl-3; β-Amylase Kit (K-BETA3)^30^ were all used following manufacturer’s instructions.

### Correlation analysis and figure preparation

All correlation and PCA analyses were carried out in RStudio using the ‘corrplot’ package. (RStudio®, Boston, USA; https://cran.r-project.org/web/packages/corrplot/corrplot.pdf). Selected graphs were prepared in SigmaPlot or Microsoft Excel. Statistical differences were determined using one-way ANOVA followed by the Tukey-Kramer test. Figures were assembled in Adobe Photoshop CS6 and Adobe Illustrator CS6.

### RNAseq analysis

Developing grain were collected from *H*. *vulgare* cv. Sloop plants at 7, 9, 11, 13, 15 and 20 days post anthesis (DPA). The embryo was discarded and all remaining (wholegrain) tissues were snap frozen in liquid nitrogen. At least six grain from three independent plants were collected and pooled to form a single composite sample at each time point. RNA for all samples was extracted using the Spectrum^TM^ Plant Total RNA kit (Sigma-Aldrich, Darmstadt, Germany). Samples were submitted for sequencing using the Illumina Hiseq Platform (AGRF, Australia), and reads were assembled against the most recent barley reference sequence using CLC Genomics^4^. Normalised read counts (transcripts per million; TPM) were determined for each HORVU sequence and used to determine the abundance of each transcript in each sample. For RNAseq analysis of pre-fertilisation stages, developing ovaries (pistils) were harvested from *H*. *vulgare* cv. Golden Promise at female gametophyte stage 4 (FG4), FG8, FG mature and FG anthesis and processed in a similar manner to that described above. The two different genotypes (Sloop and Golden Promise) were used for historical reasons; we have previously used Golden Promise as a resource for studies of floral organ fertility while Sloop has been used for studies of grain development and seedling growth.

### Laser Capture Microdissection and Quantitative PCR

Grain samples from *H*. *vulgare* cv. Sloop were collected at 11 and 25 DPA, bisected transversely and fixed in ethanol:acetic acid as described previously^31^. Tissues were embedded in butyl methyl methacrylate (BMM) and polymerised at −20 °C under UV light^31,32^. Samples were sectioned to 5 µm using a Leica Ultracut microtome, adhered to Leica PEN membrane slides and dissected using a Leica LMD microscope (Leica, Wetzlar, Germany; Adelaide Microscopy, Adelaide; Fig. S2). Approximately 6–10 sections from three grain were collected from the outer grain layers (predominantly pericarp), aleurone, outer-starchy endosperm (incorporating sub-aleurone and some adjoining starchy cells) and inner starchy endosperm (incorporating starchy endosperm cells and the grain cavity) and stored at −80 °C. Total RNA was isolated using the PicoPure kit (ThermoFisher, Australia) and converted to cDNA using Superscript^TM^ III reverse transcriptase (ThermoFisher, Australia) and oligodT primer with a 2 hour synthesis step at 37 °C. For the 25 DPA samples, RNA was amplified twice using the MessageAmp^TM^ II kit (ThermoFisher, Australia) before converting to cDNA using Superscript III and random hexamers^27^. Multiple control genes were used to normalise samples^33^ and primer sequences are included in Table S5.

## Results

### Sub-epidermal grain features are revealed by autofluorescence microscopy

The aleurone layers and cell structure present at the periphery of the barley endosperm were examined by hand-sectioning (Fig. 1A) and autofluorescence microscopy (Fig. 1B–J). UV-light revealed different types of autofluorescence depending on the filter set and clearly distinguished the pericarp (false coloured red) and aleurone cells (false-coloured yellow). Hand sections provided sufficient detail to measure transverse features of the aleurone, starchy endosperm and pericarp/husk using a 1× objective (Figs 1B–D and S1), while the number of aleurone layers and aleurone thickness could be determined using a 20× objective (Figs 1E–J and S1). To assess whether these measurements were consistent with those generated by thin sections, mature grain samples from two genotypes showing differences (Golden Promise and Flagship) were embedded in resin and sectioned prior to staining with Calcofluor White. Staining revealed differences between the genotypes, with Flagship tending to show fewer aleurone layers (Fig. S3A) than Golden Promise (Fig. S3B). A similar result was obtained by hand-sectioning (Fig. S3C,D), suggesting that the hand-sectioning method is appropriate to measure differences in aleurone phenotypes.

**Figure 2:**
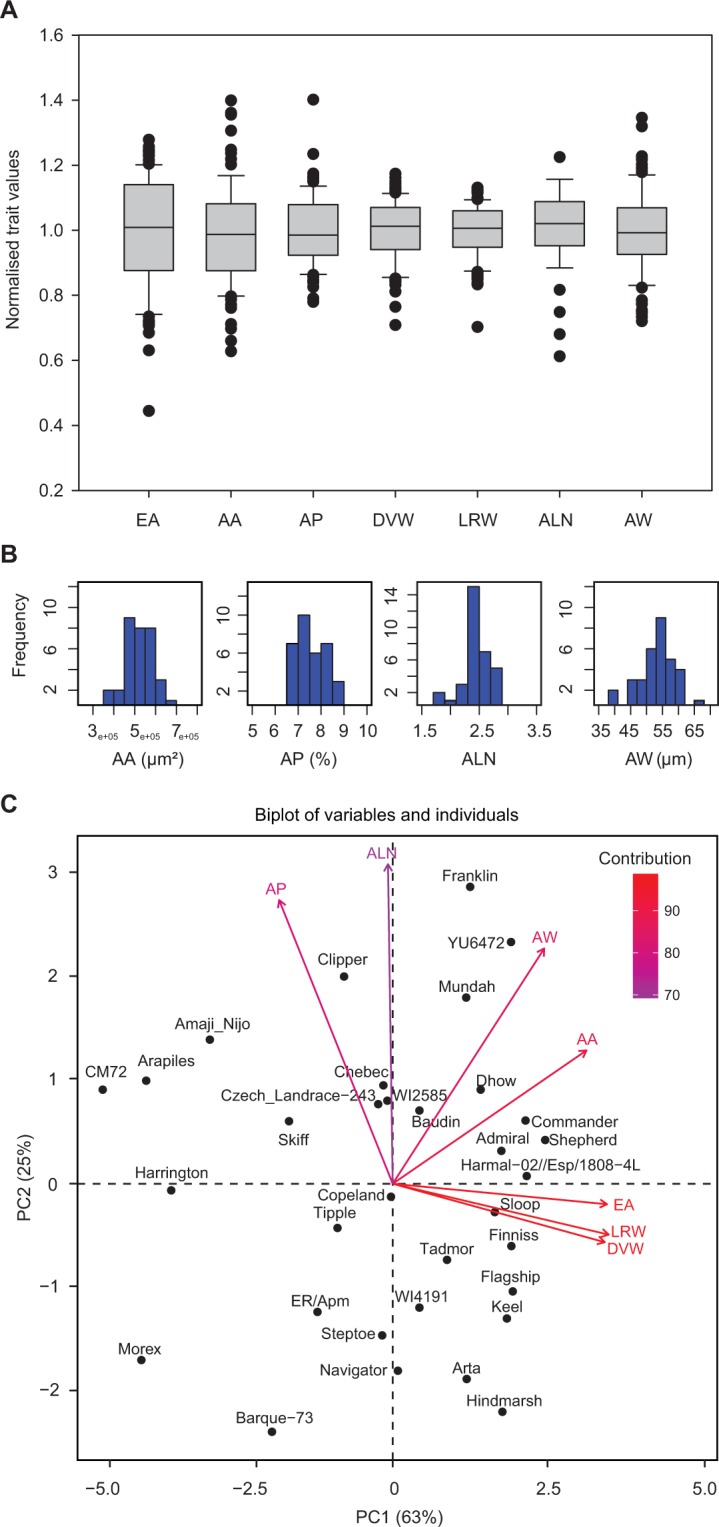
Variation in transverse grain measurements observed across 33 barley genotypes. (**A**) Box plot of normalised data showing the variation in different grain measurements. (**B**) Frequency distribution plots of the four aleurone measurements. (**C**) Principal Component Analysis separates the genotypes based on the seven transverse measurements (variables). EA, endosperm area; AA, aleurone area; AP, aleurone proportion; DVW, grain dorsal-ventral distance; LRW, transverse grain left-right width; ALN, aleurone layer number; AW, aleurone width.

Transverse grain sections were generated for 33 different barley genotypes and significant differences in aleurone phenotypes were identified (Figs 1 and 2, Table S1). Aleurone layer number was not identical around the entire grain periphery, but the average number of layers from three regions (dorsal, left and right) in each grain provided a representative measurement for comparisons between genotypes. The average number of aleurone layers for all genotypes was 2.4 ± 0.2, but showed genotype-dependent variation. Clipper (2.8 ± 0.01; Table S1) and Franklin (2.8 ± 0.01; Fig. 1E, Table S1) typically possessed more aleurone layers, Dhow showed an intermediate number of layers (2.5 ± 0.02; Fig. 1F) and Hindmarsh possessed significantly fewer layers (1.8 ± 0.05; Fig. 1G). Additionally, genotypes differed in regards to aleurone width, which was on average 53.2 ± 6.5 µm. The YU6472 genotype showed a thick aleurone (65.4 ± 6.2 µm; Fig. 1H), Mundah was intermediate (60.4 ± 4.2 µm; Fig. 1I) and Harrington possessed a thinner aleurone (40.6 ± 2.8 µm; Fig. 1J). The variation in each measurement across the panel was normalised to the average trait value, which was assigned a value of 1 (Fig. 2A). The largest variation was observed in transverse endosperm area, followed by aleurone area, aleurone layer number and aleurone width, whilst aleurone proportion and transverse grain width were less variable (Fig. 2A). The lack of variation in grain width is likely to be a result of grain screening. When focussing on aleurone-specific measurements, the values appeared to display normal distributions (Fig. 2B).

Principal component analysis (PCA) separated the genotypes based on the seven transverse grain measurements (Figs 2C and S4A). Genotypes such as CM72, Morex, Barque-73, Hindmarsh, Franklin, Harrington and YU6472 showed distinct differences.

### Differences in aleurone measurements at grain maturity correlate with other grain features

**Figure 3:**
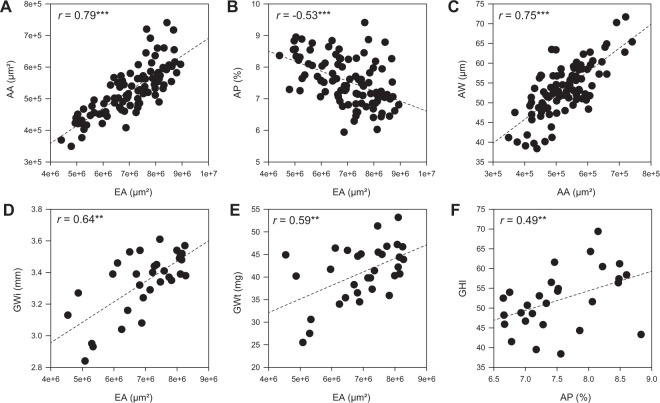
Transverse and wholegrain trait correlations across different barley genotypes. (**A**–**C**) Phenotypic measurements from all grain samples for 33 genotypes obtained using transverse grain sections. (**A**) Aleurone area vs endosperm area. (**B**) Aleurone proportion vs endosperm area. (**C**) Aleurone width vs aleurone area. (**D–F**) Correlations between grain measurements (averages) for all 2-row genotypes (n = 30) using the SeedCount, Single Kernel Characterisation System and/or transverse grain sections. (**D**) Grain width vs endosperm area. (**E**) Grain weight vs endosperm area. (**F**) Grain hardness index vs aleurone proportion. EA, Endosperm area; AA, aleurone area; AP, aleurone proportion; AW, aleurone width; GWi, grain width; GWt, grain weight. Significance indicators: ***p ≤ 0.001, **p ≤ 0.01, *p ≤ 0.05.

The relationship between mature grain and aleurone measurements was examined across the 33 different genotypes by correlation analysis (Figs 3, S4 and S5). While some traits appeared unrelated across the panel, others showed strong correlations, and in the following sections significance indicators are included as follows: ***p ≤ 0.001, **p ≤ 0.01 and *p ≤ 0.05. For example, aleurone area was positively correlated with endosperm area (0.79***; Figs 3A and S4B), while aleurone proportion negatively correlated with endosperm area (−0.53***; Figs 3B and S4B). These results suggest that although bigger grains contain more aleurone, the increase in grain size is driven by the starchy endosperm, and aleurone proliferation/expansion is compromised proportionally. Increased aleurone area was driven by increased aleurone width (0.75***; Figs 3C and S4B) but was independent of layer number, while an increased proportion of aleurone was partly due to more aleurone layers (0.41***; Fig. S4B). Aleurone width only showed a weak correlation with aleurone layer number (0.41**; Fig. S4B). Thus, larger grains contain more aleurone, mainly as a result of increased aleurone width (i.e., thickness), but smaller grains contain more aleurone layers with a higher proportion of aleurone relative to starchy endosperm. Together, these results indicate that mature aleurone morphology in barley is determined by the number and size of aleurone cells, which are in turn influenced by starchy endosperm development, and the contribution of each feature can vary depending on the genotype.

Comparisons between wholegrain and transverse section measurements were examined in greater detail for the 2-row genotypes (n = 30; Figs 3D–F and S5, Table S1). Increased transverse endosperm area was positively correlated with wholegrain measurements including grain width (0.64**; Figs 3D and S5) and grain weight (0.59**; Figs 3E and S5), confirming that some of the transverse measurements relate directly to overall grain features. Several aleurone measurements showed similar correlations to grain size; for example, aleurone area was positively correlated with grain width, thickness, area, diameter and weight (Fig. S5). Interestingly, the only transverse grain measurements to show a correlation with grain hardness were aleurone proportion (0.49**; Figs 3F and S5) and aleurone layer number (0.37*; Fig. S5). This indicates that in this panel, some 2-row genotypes producing harder grains tend to contain more aleurone layers and a higher proportion of aleurone relative to starchy endosperm.

### Differences in aleurone development are maintained across different field sites

To determine how consistent the transverse grain measurements were across different environments and years, grain from eight genotypes including Barque, Baudin, Commander, Flagship, Hindmarsh, Keel, Mundah and Shepherd were compared from Charlick, UA, in 2013 (UA), and Gooloogong, NSW, in 2015 (NVT). The aleurone and wholegrain measurements were recorded and compared as a ratio between the different environments and years, i.e., NVT value / UA value (Table S2; Fig. S6). The majority of genotypes showed less than 10% variation for all transverse measurements between environments; for example, the least variable transverse measurement was grain width (Fig. S6A) while the most variable measurements were aleurone layer number and proportion (Fig. S6A). The variation in aleurone layer number was most obvious in Barque and Hindmarsh, which tend to have more layers in the NVT samples (1.83 UA vs 2.44 NVT and 1.88 UA vs 2.38 NVT, respectively), while the variation in aleurone proportion was most obvious in Flagship, which showed more endosperm overall but less aleurone in the NVT samples. Although this reveals an effect of environment on transverse grain features, particularly with regard to the aleurone, correlation analysis indicated that the aleurone measurements generally show a similar trend between environments. For example, measurements of aleurone proportion (0.67*; Fig. S6B) and width (0.79*; Fig. S6C) were significantly correlated despite the different environments. Although this analysis is limited to a small number of genotypes, it suggests there is some degree of stability in aleurone measurements between distinct environments.

### Identification of barley genotypes for downstream analysis

Genotypes showing distinct grain phenotypes were selected for more detailed analysis based on transverse grain measurements and PCA (Figs 2C and S4A). Shepherd tended to be at the high extreme for most measurements, while Morex and Barque-73 tended to be at the low extreme (Table S1). Other genotypes were chosen to specifically examine differences in aleurone development, with the aim of avoiding confounding factors such as starchy endosperm area and grain size. For example, endosperm area in Mundah, YU6472, WI2585, WI4262 (Navigator), WI4191 and Steptoe was similar, but aleurone features such as area, proportion and width were distinct. The Mundah and YU6472 genotypes appeared to be “high” genotypes for these characteristics, WI2585 was “average”, while Flagship, WI4191 and Steptoe were “low” (Fig. 2C and Table S1). It is important to note that unlike most of the barley genotypes examined here, Morex and Steptoe are 6-row barleys. Based on the small number of 6-row genotypes in the panel, it is currently unclear whether the 6-row phenotype contributes directly to differences in aleurone development.

### Hydrolytic enzyme activities differ between genotypes with different aleurone phenotypes

**Figure 4:**
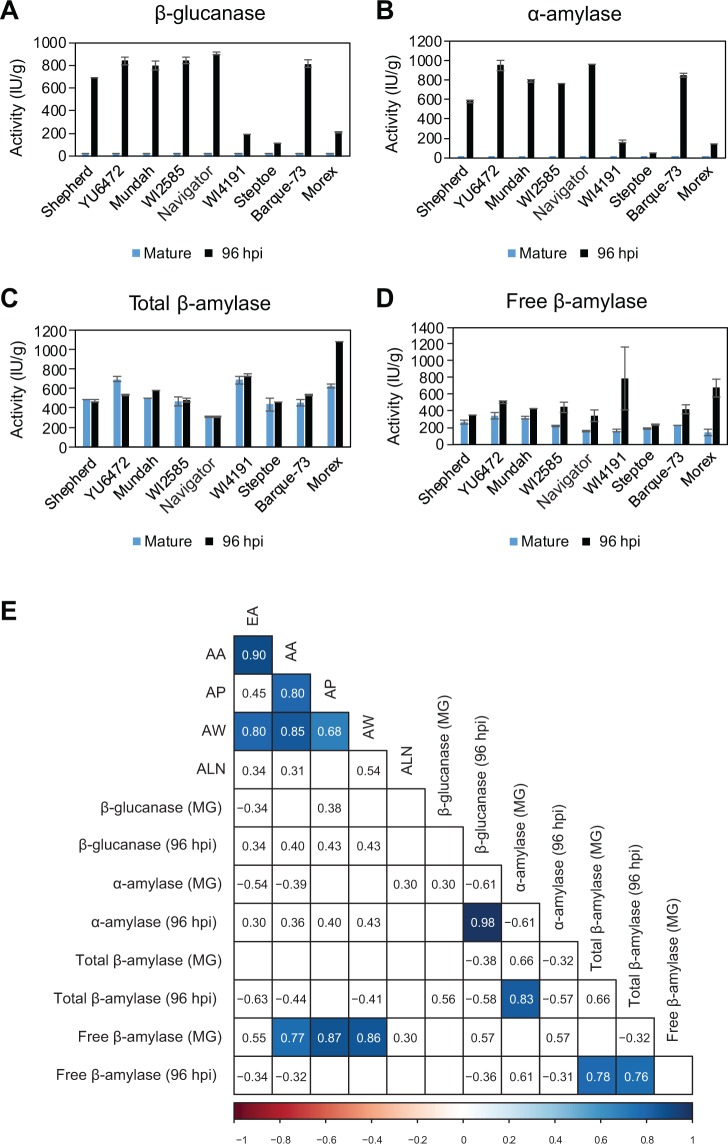
Hydrolytic enzyme activities in grain from nine barley genotypes at grain maturity and 96 hours post imbibition (hpi). (**A**) 1,3;1,4-β-glucanase (β-glucanase) activity. (**B**) α-amylase activity. (**C**) Total β-amylase activity. (**D**) Free β-amylase activity. Error bars show standard deviation. (**E**) Heat map representing correlations between aleurone measurements and enzyme activities for the nine different genotypes. Note, some aleurone correlation values differ to those in Fig. S5 due to the different sample size. Blue boxes indicate positive correlations. Numbers within boxes represent correlation coefficient (*r*) values. All values > 0.3 or < −0.3 are shown, but only those with a p-value ≤ 0.05 are contained within shaded boxes. MG, mature grain; EA, endosperm area; AA, aleurone area; AP, aleurone proportion; AW, aleurone width; ALN, aleurone layer number.

The distinct genotypes were examined to determine whether grain with more aleurone might display increased enzyme activity during germination. The results of β-glucanase, α-amylase, total and free β-amylase activity assays for the nine genotypes of interest are shown for two time points in Fig. 4, with some genotypes showing significant differences (Table S3). One of the two time points was grain maturity, allowing detection of enzymes that had been synthesised and stored during grain development, and the other was 96 hours post imbibition (hpi), which detects enzymes that have been synthesised during germination. The total β-amylase assay used a reducing agent to liberate bound enzyme before activity analysis, while the free β-amylase assay measured activity of unbound enzyme.

Consistent with previous studies, β-glucanase (Fig. 4A) and α-amylase (Fig. 4B) activity was barely detectable in mature grain, while total (Fig. 4C) and free β-amylase (Fig. 4D) activity was detectable at grain maturity. By 96 hpi, activity could be detected for all enzymes in the selected genotypes and clear differences were observed. For example, at 96 hpi, WI4191 (2-row), Steptoe (6-row) and Morex (6-row) showed relatively low β-glucanase and α-amylase activity compared to the other varieties (Fig. 4A,B). Conversely, WI4191 and Morex showed relatively high total and free β-amylase activity compared to the other varieties (Fig. 4C,D). Average total β-amylase activity was similar at grain maturity and germination for most of the varieties (Fig. 4C), although free β-amylase levels tended to be lower at grain maturity compared to the 96 hpi samples, presumably as more enzyme is released through proteolytic cleavage from starch bodies.

Correlation analysis was used to assess the relationships between aleurone morphology and enzyme activity (Fig. 4E). Significant correlations (p ≤ 0.05) were identified between (1) β-glucanase and α-amylase activity after germination (0.98*), (2) total β-amylase activity at grain maturity and free β-amylase after germination (0.78*) and (3) total β-amylase and free β-amylase after germination (0.76*). By contrast, none of the transverse endosperm or aleurone measurements showed a significant correlation with β-glucanase or α-amylase activity at maturity or 96 hpi (Fig. 4E). Also, differences in total β-amylase activity did not correlate with differences in any of the transverse grain measurements (Fig. 4E).

Conversely, free β-amylase activity showed a significant correlation (p ≤ 0.05) with transverse aleurone area (0.77*), aleurone proportion (0.87*) and aleurone width (0.86*) at grain maturity (Fig. 4E). Furthermore, although aleurone area and endosperm area were correlated (0.90*), endosperm area itself did not directly correlate with free β-amylase activity. This suggests that direct variation in aleurone cell size or area, or perhaps indirect features of the starchy endosperm that influence aleurone development, may contribute to differential abundance of free β-amylase in different barley genotypes.

To assess if variation in enzyme levels, particularly free β-amylase, might contribute to the rate of grain germination we utilised an *in vitro* germination assay (Fig. S7). Differences were observed between genotypes, particularly in the case of WI4262 (Navigator) and YU6472 (Fig. S7). Comparisons between grain features, germination frequency and enzyme levels revealed significant correlations between the number of germinated seedlings at 12 hours post imbibition (hpi) and β-glucanase and α-amylase levels at 96hpi (0.72*), and the frequency of germinated seedlings at 24 hpi and 48 hpi (0.71*). However, no significant correlation was detected between any transverse mature grain features or β-amylase activity compared to the frequency of germination at the time points analysed. Therefore, it seems unlikely that the varying aleurone features, and their association with free β-amylase activity, relate directly to the rate of germination from 12 hpi onwards.

### *β-amylase* transcript abundance varies in specific grain cell-types

**Figure 5:**
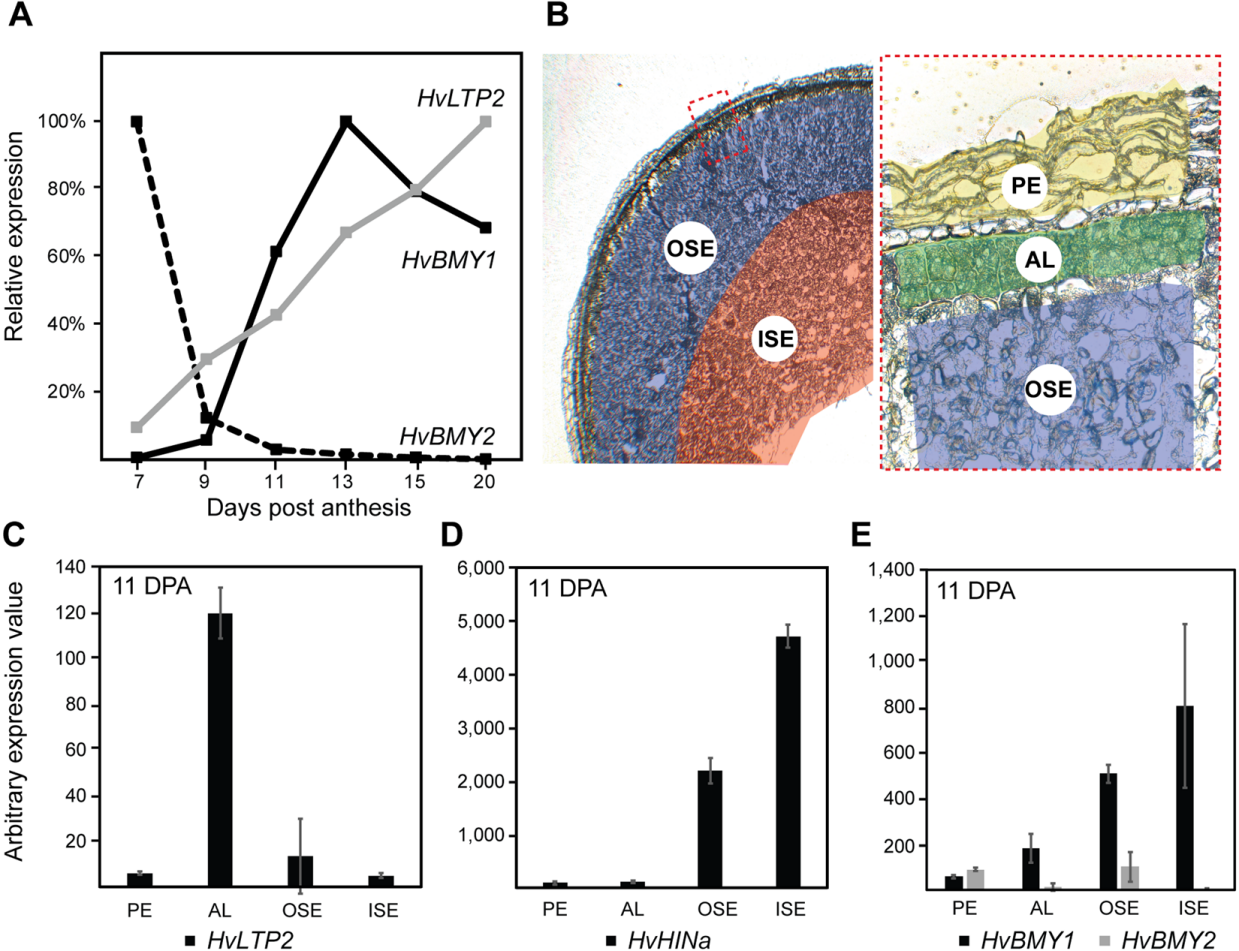
Accumulation of transcript in different barley grain tissues. (**A**) RNAseq analysis of transcripts from Sloop wholegrain samples, minus embryo, at 7, 9, 11, 13, 15, 20 days post anthesis (DPA). Accumulation patterns for the *HvBMY1*, *HvBMY2* and *HvLTP2* transcripts are shown, normalised to the maximum expression value for each gene. (**B**) Thin butyl-methyl methacrylate sections of Sloop barley grain at 11 DPA showing the regions collected by laser microdissection. PE, pericarp; AL, aleurone; OSE, outer starchy endosperm including sub-aleurone; ISE, inner starchy endosperm. The red dashed box shows a magnified view of the outer grain layers. (**C**–**E**) Quantitative PCR analysis of transcript abundance in RNA collected from laser micro dissected material. (**C**) *lipid transfer protein 2* (*HvLTP2*). (**D**) *hordoinoline a* (*HvHINa*) (**E**) *β-amylase 1* (*HvBMY1*) and *β-amylase 2* (*HvBMY2*).

To address how variation in aleurone features might directly contribute to increased wholegrain free β-amylase levels at grain maturity, we considered the spatial and temporal dynamics of *β-amylase* transcript abundance. Previous studies have shown that *β-amylase* genes are transcribed and translated during barley grain development^16,17^, with some enrichment in sub-aleurone or aleurone tissues. In this study, several datasets were generated to examine the abundance of 11 putative barley *β-amylase*-encoding genes identified in the latest release of the barley genome (Table S4). First, a developmental series of early grain development was generated from Sloop wholegrain samples (minus embryo) at 7, 9, 11, 13, 15 and 20 days post anthesis (DPA), which covers the main stages of aleurone differentiation and development (Fig. S8). This overlaps with datasets from several studies^17,34–36^. Analysis confirmed that *HvBMY1* was the most abundant *β-amylase* gene in the developing grain, increasing in abundance from 9 DPA onwards (Table S4; Fig. 5A). This pattern was distinct from that of the *lipid transfer protein 2* (*HvLTP2*) gene, a specific marker for aleurone tissue, although transcripts for both genes accumulated over time (Fig. 5A). *HvBMY2* was the second most abundant *β-amylase* transcript in the developing grain, but showed a significant decrease in abundance from 7 DPA onwards (Table S4; Fig. 5A). The expression at 7 DPA may correspond to residual expression from vegetative tissues, since in a separate dataset generated from pre-fertilisation pistil tissues (from the Golden Promise cultivar), *HvBMY2* was most abundant *β-amylase* gene (Table S4).

Next, we utilised laser microdissection to precisely separate the pericarp, aleurone, outer starchy endosperm (including sub-aleurone) and inner starchy endosperm tissues from transverse mid-point grain sections at 11 DPA (Fig. 5B) and 25 DPA (Fig. S9). RNA from these specific regions was analysed by quantitative PCR (qPCR) using known markers of grain development. At 11 DPA, transcript from the *HvLTP2* aleurone marker was barely detected in the pericarp, outer starchy endosperm and inner starchy endosperm cells, but was abundant in the aleurone (Fig. 5C). In contrast, transcript from the barley *hordoinoline* (*HvHINa*) gene that influences grain hardness and accumulates in the endosperm was predominantly detected in the outer starchy endosperm and inner starchy endosperm tissues (Fig. 5D). Unlike *HvLTP2* and *HvHINa*, *HvBMY2* transcript was predominantly detected in the pericarp and outer starchy endosperm samples, and not in the aleurone or inner starchy endosperm (Fig. 5E). *HvBMY1* transcript was detected in the aleurone, outer starchy endosperm and inner starchy endosperm samples. On average, expression was ~4 fold higher in the inner starchy endosperm tissues compared to the aleurone (Fig. 5E) and the ratio of aleurone: outer starchy endosperm: inner starchy endosperm was approximately 1:3:4. Transcript patterns were similar at 25 DPA (Fig. S9A–D), although the aleurone appeared to contribute only 3% of the total detected *HvBMY1* transcript. Taken together, these data suggest that the inner and outer starchy endosperm are the major sites of *HvBMY1* expression. However, an increase in *HvBMY1* transcript during grain development is driven by expression in multiple tissues, with the aleurone contributing up to 13% of the overall grain transcript levels depending on the developmental stage. Therefore, the increase in free β-amylase levels at grain maturity in genotypes exhibiting a larger aleurone may be partly due to expression of *HvBMY1* in aleurone tissues.

## Discussion

The cereal aleurone is a multifunctional tissue with important roles in grain development and germination, and applications in the health and brewing industries^37^. In this study, we utilised autofluorescence microscopy to identify differences in aleurone morphology within a panel of diverse barley genotypes, and considered how these differences relate to grain biology and the amount of germination-related enzyme activity.

Within the cereal grain, the aleurone and starchy endosperm are both derived from the fertilised central cell and only begin to differentiate around 7–10 days after pollination^37^. Unsurprisingly, analysis of 33 barley genotypes confirmed that development of the starchy endosperm and aleurone are intimately linked; as radial starchy endosperm area increased, so too did the radial area of aleurone. Genotypes producing grain with more aleurone layers also tended to show a thicker aleurone, and aleurone width contributed directly to aleurone area. However, the number of aleurone layers shared no direct relationship with aleurone area, suggesting that factors determining aleurone cell expansion have a greater impact on this trait. A reduced proportion of aleurone was typically linked to an increase in starchy endosperm area, while an increased proportion correlated with increased aleurone layer number. These data indicate that pathways promoting increased grain fill (i.e., starchy endosperm cell division and/or cell expansion) are (1) unlikely to be perceived by aleurone cells, (2) may inhibit the formation/maintenance of additional inner aleurone layers and (3) may indirectly impact the size of aleurone cells, possibly due to physical constraints imparted by the pericarp.

Of all the aleurone and grain features measured in this study, aleurone layer number appeared to be the most independent (see Fig. S4B). This may be due to difficulty in collecting precise measurements or perhaps a unique mechanism underlying layer formation. In maize, specific pathways appear to prevent an increase in aleurone layer number, since aberrant periclinal divisions of aleurone cells result in them adopting starchy endosperm fate^21^. A similar mechanism may contribute to subtle variations in aleurone layer number in barley. Genotypes such as Barque-73 and Hindmarsh, which produce fewer aleurone layers (~1.8 on average), may be more sensitive to differentiation signals that promote starchy endosperm identity compared to genotypes such as Clipper and Franklin, which produce more aleurone layers (~2.8 on average). The source and temporal activity of the fate-determining signals is unclear^21^; one possibility is that aleurone cells perceive a stimulatory cue at the periphery of the grain that only reaches a certain radial depth. Similar basic mechanisms have been identified in Arabidopsis, where diffusible epidermal signals control sub-epidermal cell identity during shoot meristem development^31,38^. Alternatively, the starchy endosperm generates signals that do not reach or are not perceived by the aleurone. The diversity of aleurone phenotypes observed in this barley panel provides an opportunity to address these differences at the genetic level in future studies.

### The contribution of sub-epidermal tissues to wholegrain traits is revealed by transverse sectioning and microscopy

One limitation of manual microscopic screens is their low throughput nature, particularly compared with high throughput automated screens of grain shape, composition and dimension used in breeding programs. However, the advantage of microscopy is that sub-epidermal features of the grain can reveal cell-type specific contributions to wholegrain traits^39^. Here, comparison of microscopy and wholegrain analyses showed clear correlations, particularly when focussing on spring 2-row barley genotypes. For example, transverse endosperm area correlated with wholegrain measurements of area, thickness and weight (Fig. S5).

Unexpectedly, grain hardness correlated positively with aleurone proportion and layer number (Fig. S5). Grain hardness has been intensively studied in the cereals. In wheat, hardness contributes to milling and baking properties and flour composition^40–43^, while in barley, hardness influences pearling properties^44^ in addition to the malting quality index^45^. The composition of individual grain components, particularly the starchy endosperm, determines whether the grain will be hard or soft^45–47^ with models suggesting that harder grains have a denser endosperm with a continuous protein matrix that prevents easy release of starch granules^39^. A specific relationship between the barley aleurone and grain hardness does not appear to have been reported previously. The correlation detected here may therefore reflect an effect of starchy endosperm protein on hardness, and an indirect effect on aleurone development. Another possibility is that in the examined panel, differences in the chemical and physical properties of the aleurone cell walls may directly contribute to grain hardness. Barley aleurone cell walls are enriched in arabinoxylan polysaccharides cross-linked with phenolic acids such as ferulic acid^12,14^, forming a robust matrix that surrounds the grain during development^37^. In genotypes with an increased proportion of aleurone, this reinforced cell wall matrix may provide a harder shell around the grain. This is something that might also be considered in future studies.

### Variations in barley aleurone features provide opportunities for further genetic analysis, and do not appear to impact overall grain development

Despite several studies providing insight into the genetic architecture of barley aleurone development^23,48^ the molecular basis for variation between genotypes has yet to be elucidated. Genetic data is available for a number of the genotypes investigated here, but the number was insufficient to carry out a robust genome wide association study (GWAS) to identify possible quantitative trait loci (QTL). However, our findings show that the variation between genotypes is reproducible and statistically significant; this suggests that a similar screen might be carried out on a larger panel of genotypes to support future genetic analysis.

Strangely, it has also remained unclear whether intraspecific differences in barley aleurone development are of any physiological importance. It is possible that the variation is of no major consequence, as long as the aleurone is still present and able to fulfil roles in hormone perception and enzyme release during germination. In general, mutants that show a lack of or reduced number of aleurone layers tend to show defects in seed development. For example, the *barley defective seed 5* mutant (*des5*)^48^ shows a patchy reduction in the number of aleurone cells, and severe defects in starchy endosperm fill and seed morphology. Similarly, mutations in the maize *naked endosperm1* and *crinkly4* genes lead to reduced aleurone phenotypes, in addition to compromised whole seed morphology^24,27^. On the other hand, mutant alleles of the maize *supernumerary aleurone 1* (*sal1*) gene, which produce two or three aleurone layers instead of the one layer detected in wild type, have relatively normal kernels^26,49^. Variations in aleurone thickness, area and layer number from two to four layers appeared to have no detrimental impact on overall grain development across the barley panel examined here.

The genotypes investigated included a combination of 2-row and 6-row varieties, malting and feed varieties; for example, Barque-73 and Mundah are Australian feed varieties, Sloop is an Australian malting variety, and YU6472 is a Chinese feed variety^50^. Based on an “average” sized grain, Barque-73 exhibited reduced aleurone area, proportion and width. At the other extreme, compared to its average grain size, YU6472 displayed increased aleurone area, proportion and width. Barque-73 also showed significantly fewer aleurone layers compared to Mundah and Sloop. Although the sample size is small, there appeared to be no clear difference in aleurone morphology to distinguish between grains from feed and malting genotypes. This observation needs to be treated with some caution, however, since there are many features that contribute to malt grade barley^51^. This could be tested in a larger panel of genotypes that have been directly assessed, head-to-head, for malt quality.

### Genotypes with more aleurone show increased levels of free β-amylase at grain maturity

The variation present in this barley panel provided an opportunity to assess the effect of different aleurone phenotypes on the activity of germination-related enzymes, which is one important aspect of aleurone function. During barley grain imbibition, gibberellic acid (GA) is released by the scutellum, triggering the synthesis and subsequent release of various hydrolytic enzymes from the aleurone^52–54^. Of these enzymes, β-glucanase facilitates the hydrolysis of β-glucan polysaccharides in cell walls and allows access to starch for additional hydrolytic enzymes^55^. Enzymes involved in starch hydrolysis include α-amylase, which hydrolyses α-1,4-glycosidic bonds of starch polysaccharides and β-amylase, which is synthesised during grain development, and during germination acts to liberate the disaccharide maltose from the non-reducing end of starch molecules^16,56–58^. Function of these enzymes is critical for germination^59,60^, and previous studies show that mature grain β-amylase content varies between barley genotypes^61–63^.

β-glucanase enzyme activity was barely detectable at grain maturity, but was high at 96 hpi, and the same pattern was observed for α-amylase. A relatively low level of activity was identified for both enzymes in WI4191, Steptoe and Morex, which tend to display “low” aleurone phenotypes. However, neither β-glucanase nor α-amylase levels showed a general correlation with differences in mature grain aleurone morphology. This may indicate that variation in transverse aleurone morphology at maturity has no direct impact on the amount of β-glucanase and α-amylase activity. Alternatively, the genotypes chosen for analysis did not show large enough differences in aleurone development, the panel was too small, or the 96 hpi time point was too late to identify such differences.

Two forms of β-amylase are present in the grain, a bound and free form. Bound β-amylase is located in an insoluble protein complex, mainly associated with the periphery of starch granules via disulphide bridges^18,64^, while the soluble or free form is active. Both forms of β-amylase are identical in terms of mobility and molecular specific activity, indicating that once bound β-amylase is cleaved, it is converted to free β-amylase^18^. Total and free β-amylase were detected in mature and germinated grain samples from the nine genotypes of interest. Total β-amylase activity did not change over time, consistent with its synthesis during grain development. Free β-amylase activity increased during germination and varied between genotypes, consistent with the release of bound β-amylase and previous reports^16,19,61–63^. Notably, genotype-specific differences in free β-amylase activity at grain maturity correlated with aleurone area, proportion and width. Genotypes with “high” aleurone phenotypes exhibited higher free β-amylase levels. In physiological terms, we propose this may allow for an early pulse of starch hydrolysis prior to the liberation of bound β-amylase by endopeptidases. In wheat, hydrogen sulphide treatment was shown to stimulate early germination through early activation of β-amylase^65^. The authors speculate that higher levels of “active” free β-amylase can participate in starch hydrolysis, providing sugar units for seedling growth prior to the induction of α-amylases by GAs^65^. In the current study, a role for increased free β-amylase activity in germination was tested in the context of nine genotypes of interest, but this failed to identify any significant correlation. This suggests that if there is a physiological role for increased free β-amylase levels and aleurone features at maturity, it may occur prior the emergence of the barley radicle at 6 to 12 hpi.

### Explaining differences in free β-amylase levels at grain maturity

There are a number of reasons why free β-amylase levels might vary at grain maturity, including variable transcriptional dynamics of different *HvBMY* genes and polymorphisms that influence enzyme activity. Previous studies indicate that at least two β-amylase genes are expressed during grain development, and that *HvBMY1* rather than *HvBMY2* is likely to be the most important *β-amylase* gene involved in germination^66^. Our RNAseq data and analysis of 11 *HvBMY* genes from the Sloop cultivar supports this finding (Table S4). Moreover, many of the cultivar-specific differences in total and free β-amylase levels can be explained by cultivar-specific differences in the *HvBMY1* gene^51,61,67,68^. In the Chebec and Harrington cultivars, the *HvBMY1* locus on 4HL accounts for approximately 90.5% of the variation in free β-amylase levels^62^. Barley genotypes can be of the Sd1-type (Harrington; lower free β-amylase levels) or Sd2-type (Chebec; higher free β-amylase levels), and this is attributed to distinct amino acid substitutions in *Hv*BMY1. In addition, differences in intron 3 of the *HvBMY1* gene, a possible site of *cis*-regulatory-elements, may contribute to differences in total β-amylase levels^69^. Furthermore, results from an earlier study^18^ suggest that grain desiccation may also impact free β-amylase levels, since it contributes to the process of β-amylase being bound to starch.

Based on the well-characterised function of β-amylase post-germination, is seems unlikely that different *HvBMY* genes or alleles contribute directly to differences in aleurone morphology during early grain development. Rather, we hypothesise that differences in aleurone development may be another factor that impact free β-amylase levels at grain maturity. This hypothesis is supported by several findings. First, early studies indirectly suggested the presence of β-amylase enzyme in the aleurone layer^20^ and the sub-aleurone layer^64,70^. Second, genes encoding β-amylase are transcribed in the aleurone. In the Barke cultivar, *HvBMY1* is the most abundant gene family member expressed during grain development, and was detected in the aleurone and sub-aleurone by mRNA *in situ* hybridisation^17^. In the same study, *HvBMY2* was detected at low levels in the endosperm, but was most abundant in the pericarp where it peaked at 6 DPA^17^. Our results in Sloop wholegrain for *HvBMY1* and *HvBMY2* show a similar temporal pattern and relative abundance during grain development, indicating that *HvBMY1* transcript is highly abundant when the aleurone is forming. Third, studies in a number of temperate grasses including barley show that the aleurone is essentially free of starch granules^71^, suggesting it may provide a starch-free repository for free β-amylase storage.

In the genotypes investigated here, approximately half (on average 45 ± 14%) of the total β-amylase appears in a free form at grain maturity. Based on the relative abundance of *HvBMY1* transcript in different grain compartments, it seems unlikely that all of the free β-amylase is derived solely from the aleurone. In the Sloop cultivar, laser microdissection qPCR revealed that *HvBMY1* is detected in the aleurone, outer starchy endosperm (incorporating the sub-aleurone) and inner starchy endosperm cells. At 11 DPA, when *HvBMY1* transcript levels are increasing in the grain, approximately 13% of transcript is derived from the aleurone. If all of this transcript is translated directly into β-amylase and remains unbound (free) due to the absence of starch, then the aleurone would contribute ~30% of the free β-amylase activity detected at grain maturity. Hence, variation in the amount of aleurone between cultivars could potentially contribute to the variation observed in free β-amylase activity, but it is clearly not the main determinant.

Several points need to be considered in future studies. It is currently unclear whether *HvBMY1* transcript abundance varies along the length of the barley grain, particularly near the embryo, which may lead to an underestimation of aleurone *HvBMY1* levels. It is also possible that the relative abundance of aleurone *HvBMY1* transcript peaks at a time point that was not investigated here (for example 13 DPA where wholegrain *HvBMY1* levels peak), before they decrease at 25 DPA. Along these lines, it is unclear exactly when the differences in aleurone development are manifested in the examined genotypes. If the differences appear early, coinciding with the stage where *HvBMY1* transcript is most abundant, then this may have an impact on downstream *Hv*BMY1 levels. Finally, antibodies to β-amylase have been reported^72^, but current microscopic assays that distinguish the different β-amylase forms are unavailable and need to be established. These would be useful tools in determining the location of the enzymes, assessing variation between genotypes of interest and determining the dynamics of enzyme release during seed development and germination.

## Electronic supplementary material

Supplementary Figures

Supplementary Tables
