# Supplementary material for: Differences in hydrolytic enzyme activity accompany natural variation in mature aleurone morphology in barley (Hordeum vulgare L.)
Source: Sci Rep. 2018 Jul 23;8:11025. doi: 10.1038/s41598-018-29068-4 (PMC6056469; doi:10.1038/s41598-018-29068-4)
Supplement: Supplementary file 1 — Supplementary Figures [file 41598_2018_29068_MOESM1_ESM.pdf]

Supplementary Data

Differences in hydrolytic enzyme activity accompany natural variation in mature aleurone morphology in barley (*Hordeum vulgare* L.)

Matthew K. Aubert, Stewart Coventry, Neil J. Shirley, Natalie S. Betts, Tobias Würschum, Rachel A. Burton and Matthew R. Tucker

Figure S1

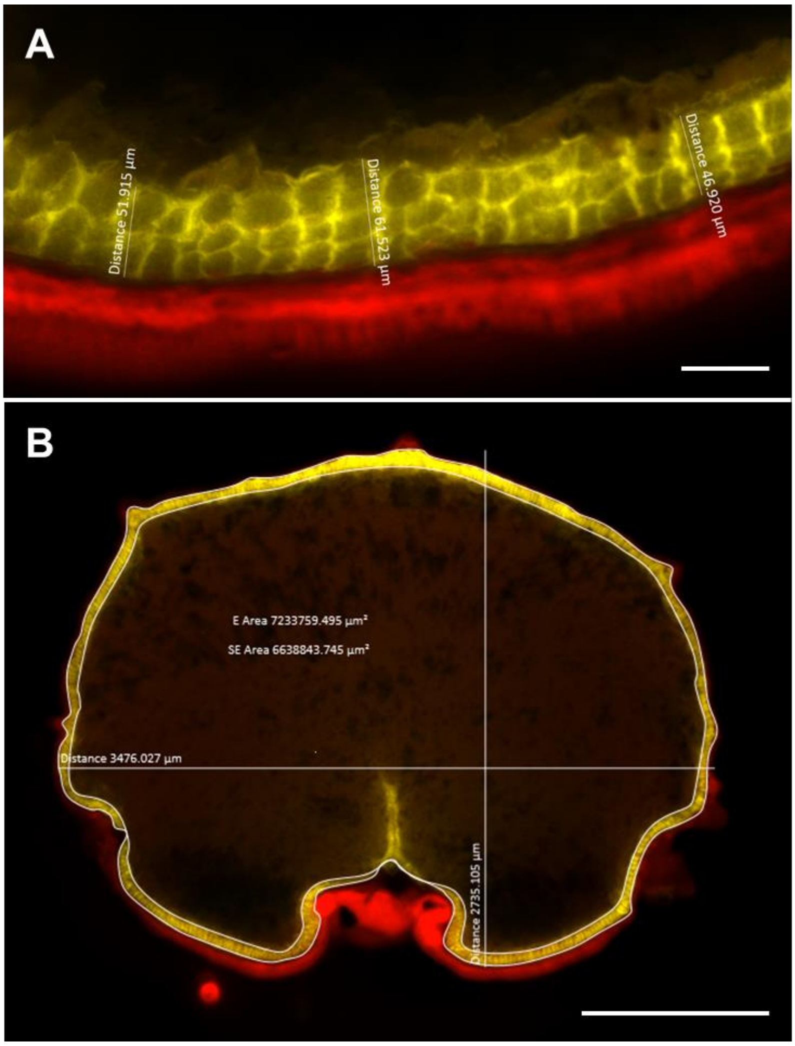

Figure S1: Transverse grain measurements were recorded using ZEN 2012 software. Grain were prepared as shown in Fig. 1. (**A**) Image at 20× magnification showing aleurone thickness (width) measurements. (**B**) Image at 1× magnification showing transverse grain measurements such as endosperm (E) area, starchy endosperm (se) area, grain thickness and grain width. Scale bar = 50 µm in A and 1mm in B.

Figure S2

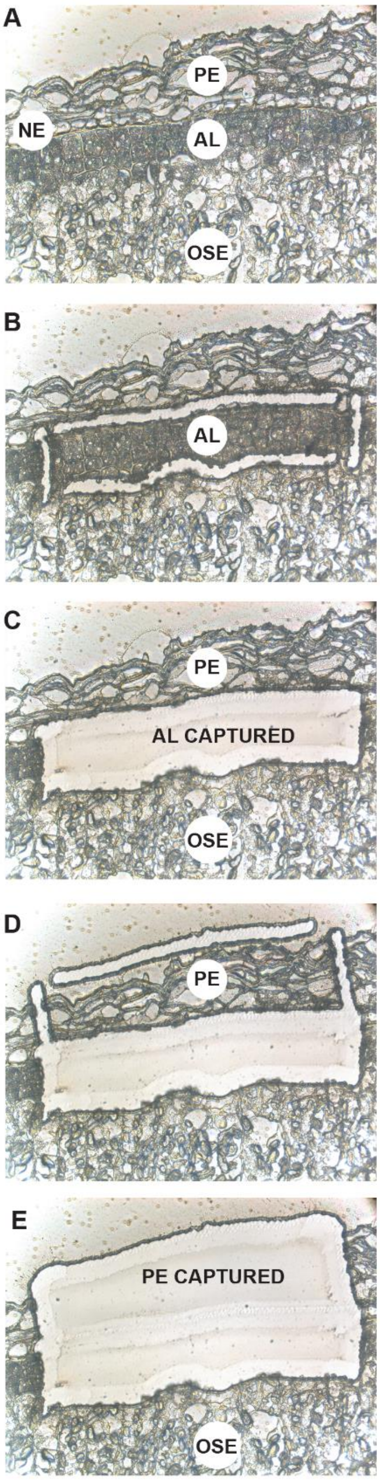

Figure S2: Barley grain tissues harvested by laser microdissection. A 25 DPA grain sample is shown. (**A**) A butyl-methyl methacrylate (BMM) section (5 µm) showing the outer layers of

the grain prior to dissection. PE, pericarp; NE, nucellar epidermis; AL, aleurone; OSE, outer starchy endosperm including the sub-aleurone. (**B-C**) Laser ablation of cells directly adjoining the aleurone prior to capture. (**D-E**) Collection of the outer maternal grain layers.

Figure S3

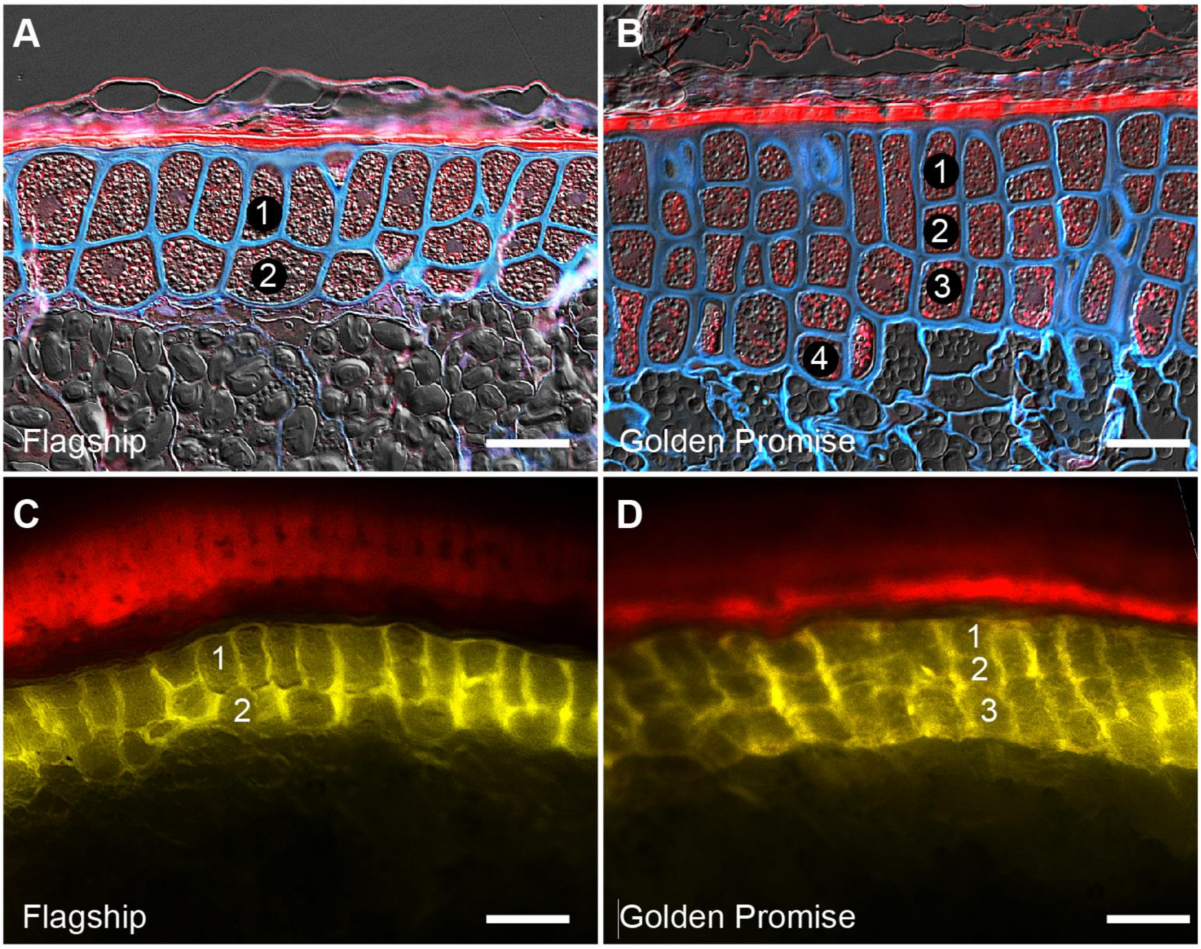

Figure S3: Comparison of sectioning methods for the investigation of mature barley aleurone morphology. (**A-B**) Thin 1 µm sections of mature grain from Flagship and Golden Promise cultivars stained with Calcofluor White (blue). The pericarp and aleurone grains show autofluorescence using Zeiss Filter set 46 (false-coloured red). The aleurone in Flagship contains 2-3 cell layers, while the aleurone in Golden Promise contains 3-4 aleurone layers. (**C-D**) Hand sections of barley grain from the same cultivars shown in (**A-B**). The pericarp is detected using Zeiss Filter set 46 (false-coloured red) and the aleurone is detected using Filter Set 49 (DAPI; false-coloured yellow). The aleurone layer number detected by thin sectioning (shown in **A** and **B**) is the same as that detected by hand sectioning without staining. Scale bar = 50 µm in A and B, 60 µm in C and D.

Figure S4

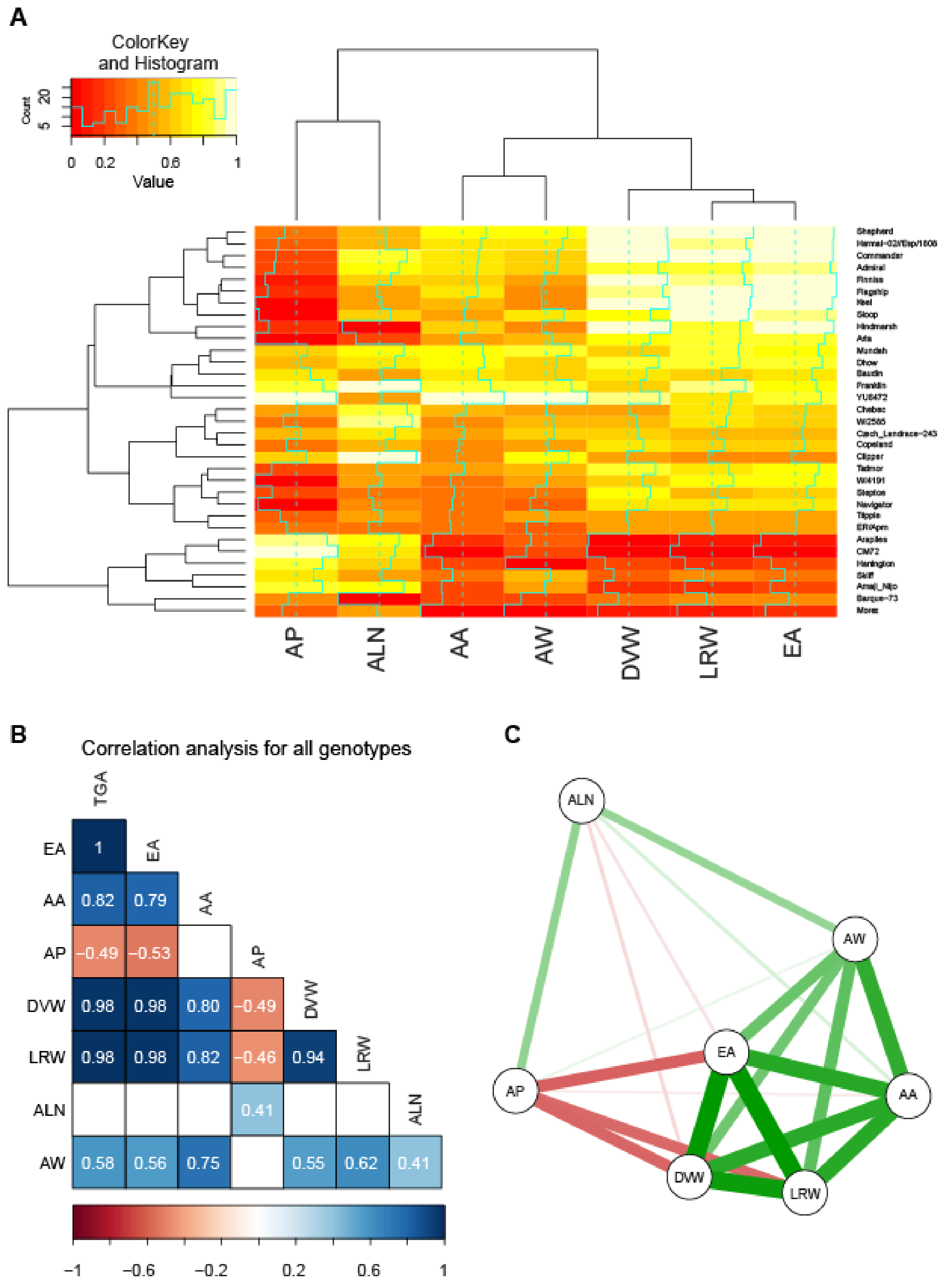

Figure S4: Relationships between transverse grain measurements in a panel of 33 barley genotypes. (**A**) Heatmap showing clusters of different genotypes separated based on the seven different grain measurements. Trait values are normalised to a value between 0 and 1

and the blue line indicates the ranking of each genotype for each measurement. (**B**) Heatmap representing correlations between transverse grain measurements for 33 barley genotypes. Blue boxes indicate positive correlations and red boxes indicate negative correlations. Numbers within boxes represent correlation coefficient (*r*) values and only those correlations with a p-value ≤ 0.05 are shown. TGA, transverse grain area; EA, starchy endosperm area; AA, aleurone area; AP, aleurone proportion; DVW, grain dorsal-ventral width; LRW, grain left-right width; ALN, aleurone layer number; AW, aleurone width. (**C**) Network analysis of the transverse grain measurements according to correlations shown in **B**. Line thickness provides an indication of the strength of correlation, with green indicating a positive correlation and red indicating a negative correlation.

Figure S5

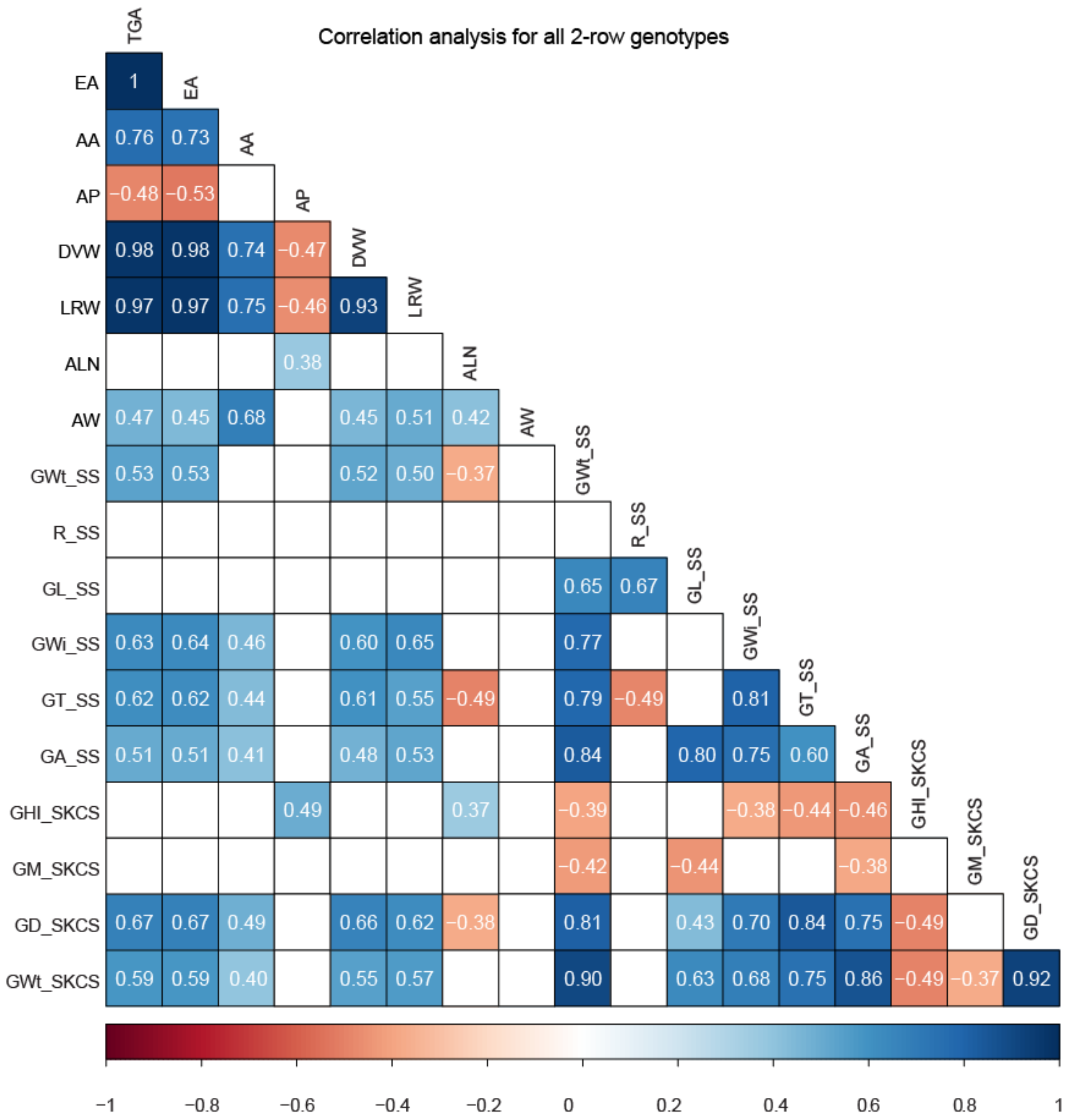

Figure S5: Correlations between aleurone and wholegrain measurements for the 30 2-row barley genotypes. Blue boxes indicate positive correlations and red boxes indicate negative correlations. Numbers within boxes represent correlation coefficient (*r*) values and only those correlations with a p-value ≤ 0.05 are shown. TGA, transverse grain area; EA, starchy endosperm area; AA, aleurone area; AP, aleurone proportion; DVW, grain dorsal-ventral width; LRW, grain left-right width; ALN, aleurone layer number; AW, aleurone width; GWt, grain weight; R, grain roundness; GL, grain length; GWi, grain width; GT, grain thickness; GA,

grain area; GHI, grain hardness index; GM, grain moisture; GD, grain diameter; SS, seed scanner; SKCS, single kernel characterisation system.

Figure S6

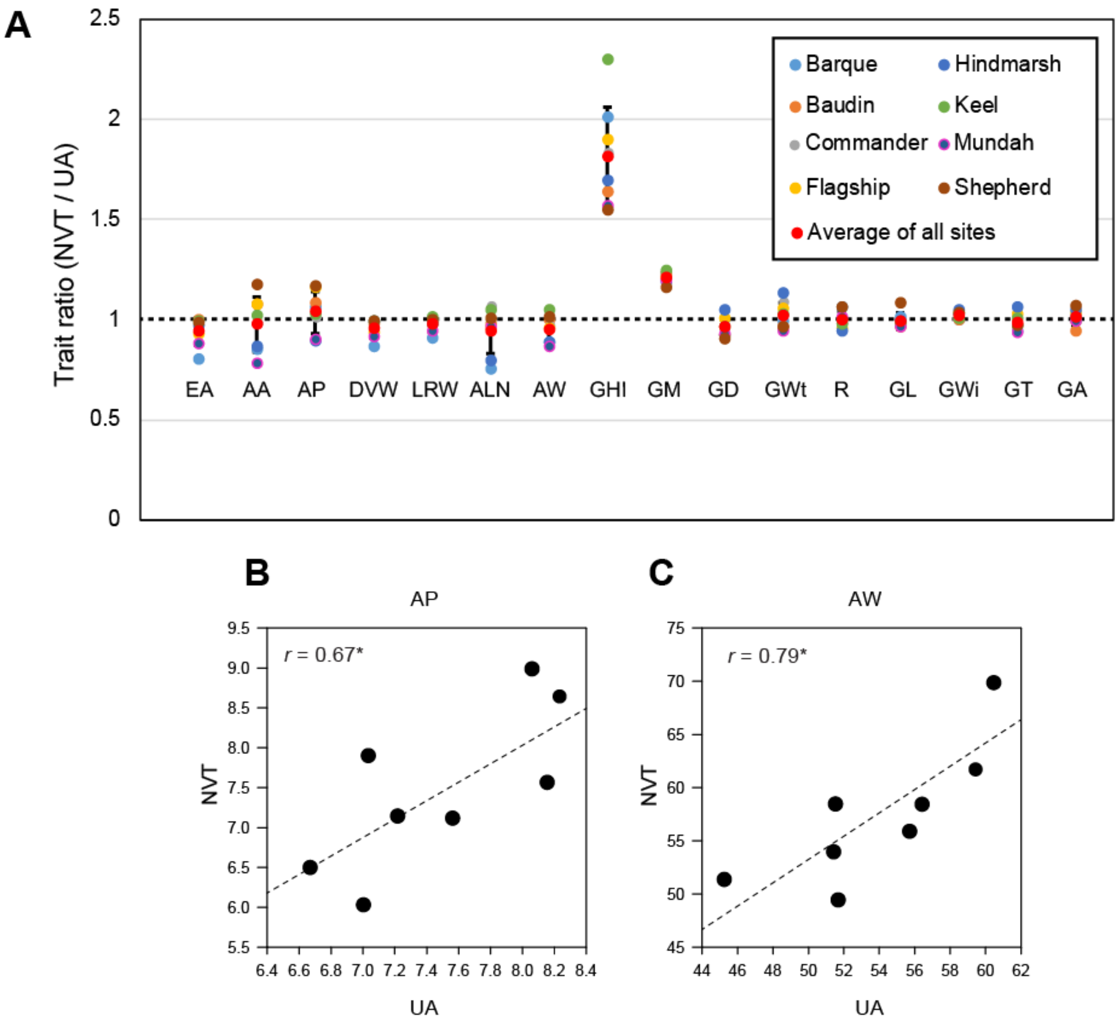

Figure S6: Comparison of grain measurements for eight barley genotypes grown in field sites at Charlick SA (2013) and Goologong NSW (2015). (**A**) The Goologong samples formed part of the National Variety Trials (NVT) and measurements are shown as a proportion of the University of Adelaide panel (UA) value for each genotype. A value of 1 indicates the measurement was identical in the different panels. Error bars show standard deviation. (**B–C**) Scatterplots showing the correlations for eight genotypes from the UA and NVT panels. The correlation coefficients (*r*) are shown. EA, endosperm area; AA, aleurone area; AP, aleurone

proportion; DVW, grain dorsal-ventral width; LRW, grain left-right width; ALN, aleurone layer number; AW, aleurone width; GHI, grain hardness index; GM, grain moisture; GD, grain diameter; GWt, grain weight; R, grain roundness; GL, grain length; GWi, grain width; GT, grain thickness; GA, grain area. Significance indicators: * = $p \leq 0.05$.

Figure S7

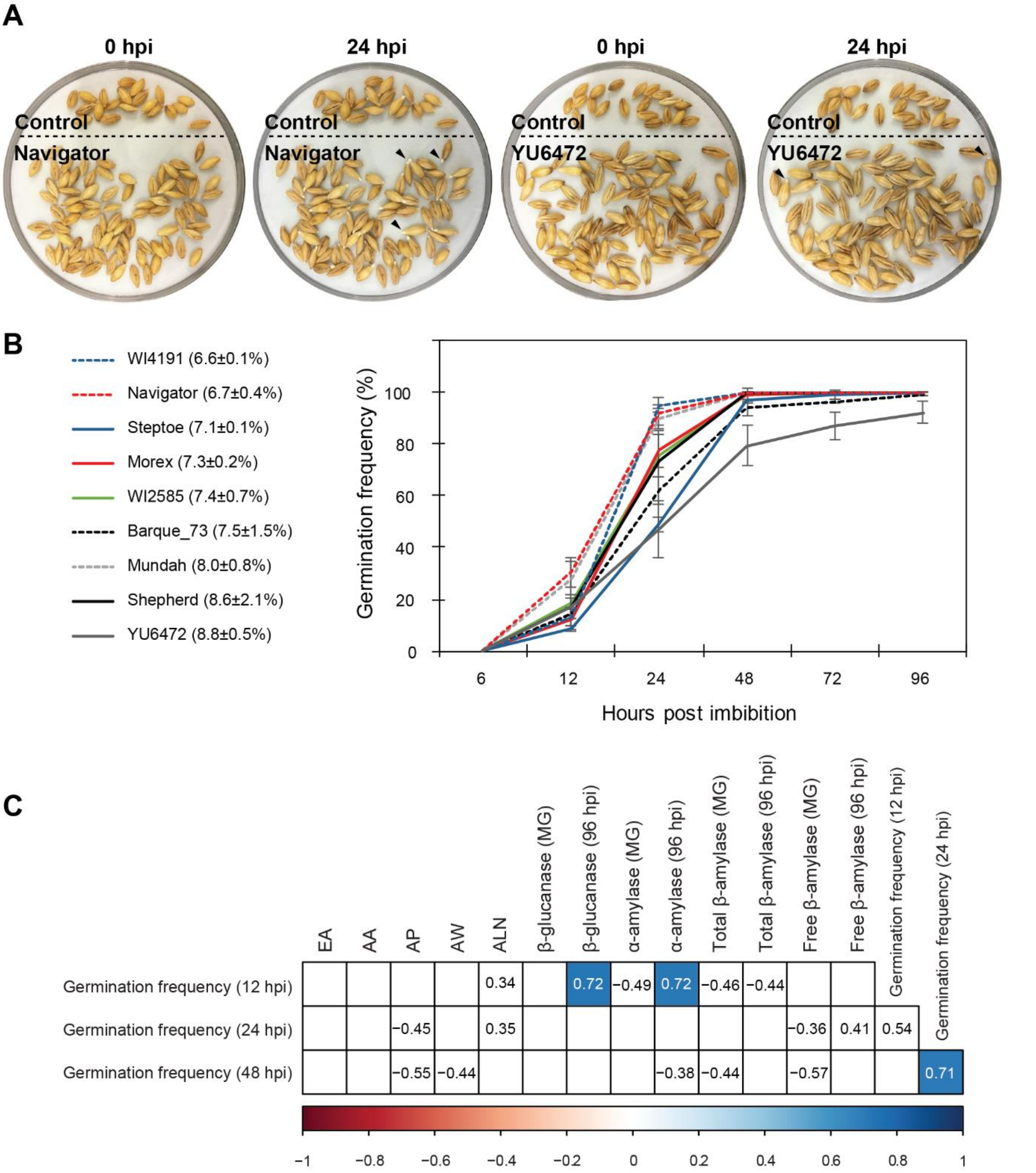

Figure S7: Analysis of germination frequency in nine barley cultivars showing distinct aleurone features. (**A**) Petri dishes containing grains 0 hours post imbibition (hpi) and 24 hpi for the Navigator and YU6472 genotypes. Control grain are from the Flagship genotype. Arrowheads indicate the emergence of the radicle. (**B**) Frequency of germination at six time points after imbibition. The legend shows the cultivars sorted from smallest to largest with regards to aleurone proportion. Error bars show standard deviation. (**C**) Correlation matrix for

germination frequency, aleurone features and enzyme activity levels. Correlation coefficients greater than 0.3 or less than -0.3 are shown, but only coloured squares indicate a p-value ≤ 0.05.

Figure S8

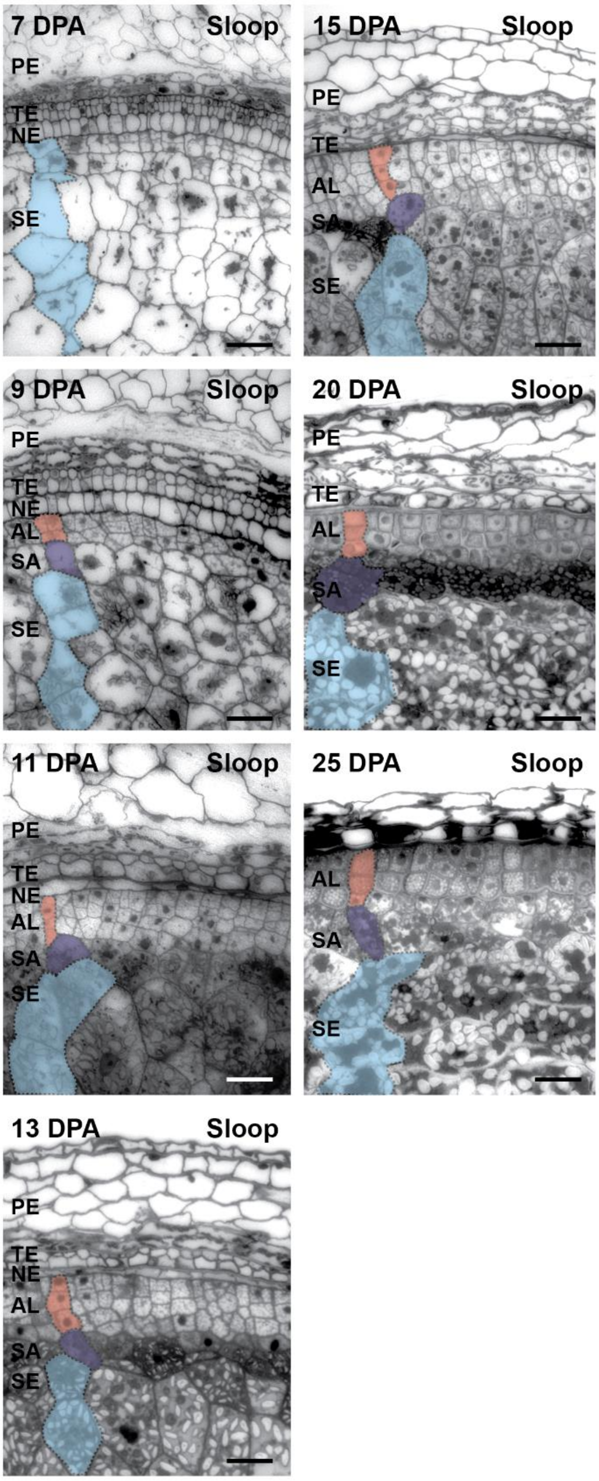

Figure S8: Stages of grain development from Sloop, corresponding to those collected for RNAseq analysis. Developing grain were embedded in LR-white and sectioned (1μm) prior to

staining with Toluidine blue. Selected aleurone cells are false coloured in orange, sub-aleurone cells in purple and starchy endosperm cells in blue. DPA, days post anthesis; AL, aleurone; NE, nucellar epidermis; PE, pericarp; SE, starchy endosperm; SA, sub-aleurone; TE, testa (integuments). Bar = 50µm.

Figure S9

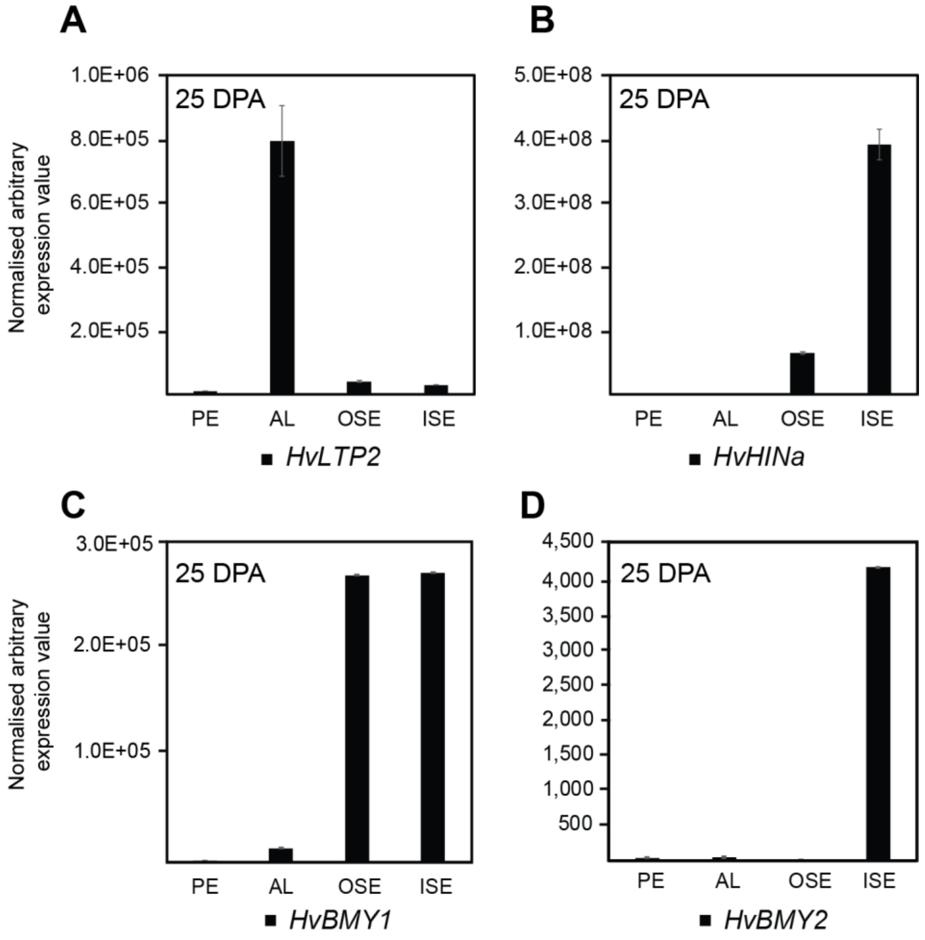

Figure S9: Transcript analysis in grain tissues harvested by laser capture microdissection at 25 days post anthesis (DPA). In contrast to Fig. 5, captured RNA was amplified before conversion to cDNA. Quantitative PCR analysis of transcript abundance was undertaken for (**A**) *lipid transfer protein 2* (*HvLTP2*), (**B**) *hordoinoline_a* (*HvHINa*), (**C**) *β-amylase 1* (*HvBMY1*) and (**D**) *β-amylase 2* (*HvBMY2*). PE, pericarp; AL, aleurone; OSE, outer starchy endosperm including sub-aleurone; ISE, inner starchy endosperm.
